# Mastitis Treated with Phytolacca Decandra

**Published:** 1875-09

**Authors:** T. Curtis Smith

**Affiliations:** Middleport, Ohio


### MASTITIS TREATED WITH PHYTOLACCA DECANDRA.

BY T. CURTIS SMITH, M. D., MIDDLEPORT, OHIO.

Several years ago my attention was called to the use of phytolacca, in the treatment of mammary abscess, but being somewhat skeptical in regard to its value, I declined using it until I had failed to prevent suppuration in several cases, by any treatment I had been able to bring to bear upon them. Finally, Drs. Tidd and Bishop, of this place, called especial atttention to it as a valuable agent for this purpose. Having a case on hand, in a broken down woman, who was well worn out with hard work and frequent parturitions, and, who had, at every confinement for the last four years, suffered from mastitis, and having once, in her case, failed to abort it, and seeming very likely to do so again, I put her on the use of fifteen-drop doses every four hours of the saturated ticture. I had for four days, previous to its use, tried various agents in vain, to allay the inflammation, and had not much faith in the value of this one. In twenty-four hours from the first dose, there was an evident improvement in the condition of the gland, and in a very few days all evidence of the threatened abscess had entirely disappeared. In this instance the secretion of milk ceased entirely, a result much desired, but not expected from this agent. True, such might have been the case any hour, as a consequence of her being so much debilitated and the blood so much impoverished.

A short time after this, a stout, healthy young married lady was prematurely confined, and from death of the fœtus, it became necessary to draw off the overflow of milk, which came on abundantly in her glands. From imprudence and failure to keep the milk drawn out, an abscess threatened severely for two or three days before my attention was called to it. Upon visiting her, and inspecting the threatened gland, I found it greatly enlarged, hard, and nodulated; she was feverish, had been chilly several times, and sweated profusely afterwards. I ordered the phytolacca, as in the previous case, applying it also to the breast. At my visit the next day I was very much surprised to find that the inflammation had so much subsided as to scarcely deserve further attention, the breast being soft and but little tender. In this case, although the milk did not dry up as promptly as in the previous case, the agent seemed to exert a powerful influence in diminishing the secretion. It is not at all improbable that the abscess in this case could have been prevented by other means, but certainly few remedies could apparently have accomplished the end desired, with more promptness than did the agent in question.

A few months since, I attended a young married lady in her confinement—primepara—the infant being still-born, apparently having been dead for a considerable time. At the usual time, after her confinement, the milk appeared in great abundance, and was kept clear by light, careful pumping, and by running out spontaneously. But, from some cause beyond my search, she suddenly had a chill, followed by fever and profuse sweating. The attack I looked upon as probably ephemeral in character; gave a saline cathartic and febrifuge, and left, requesting to be called by the second day if she did not rapidly improve. Being called, I found her with both mammary glands very largely swollen, hard, nodulated, extremely tender and painful. I at once ordered the phytolacca, as in former cases, but every three hours, at the same time giving quinia freely to combat a periodic return of the fever, which had put in an appearance every evening. The next evening all of her untoward symptoms had abated to a considerable extent, and by the fourth day, the fever failed to return, and the mammary glands were comparatively soft and free from pain. The case went on to complete recovery without further trouble. In other cases of this type it has always acted very well, not having failed, as yet, in any case where its use was resorted to before positive evidence of suppuration had occurred.

Whether this agent would prove of value, in preventing the formation of abscess in other glands, or in other tissues, I am not prepared to state from experience, but by inference it seems worthy of the trial. The milk in the last case dried up rapidly after using the remedy. Its use in other affections has proved it to be a very valuable vegetable alterative. That it has any special mode of action upon the economy, other than as an alterative, I cannot say. I have used it, so far, empirically with the results above given. In muscular rheumatism, it is a very good remedy, often affording marked relief in a few hours.

In giving the agent, it should be remembered that in over doses it is a powerful gastric irritant, the nausea and vomiting provoked by it being difficult to control, the prostration being very marked.

